# Regulation of Aquaporin Functional Properties Mediated by the Antioxidant Effects of Natural Compounds

**DOI:** 10.3390/ijms18122665

**Published:** 2017-12-08

**Authors:** Giorgia Pellavio, Marta Rui, Laura Caliogna, Emanuela Martino, Giulia Gastaldi, Simona Collina, Umberto Laforenza

**Affiliations:** 1 Department of Molecular Medicine, Human Physiology Unit, University of Pavia, I-27100 Pavia, Italy; giorgia.pellavio@gmail.com (G.P.); gastaldi@unipv.it (G.G.); 2 Department of Drug Sciences, Medicinal Chemistry and Pharmaceutical Technology Section, University of Pavia, I-27100 Pavia, Italy; marta.rui01@universitadipavia.it (M.R.); simona.collina@unipv.it (S.C.); 3 Operative Unit of Orthopaedics and Traumatology, Fondazione IRCCS Policlinico San Matteo, I-27100 Pavia, Italy; L.Caliogna@smatteo.pv.it; 4 Department of Earth and Environmental Sciences, University of Pavia, I-27100 Pavia, Italy; emanuela.martino@unipv.it

**Keywords:** water channel, oxidative stress, antioxidant compounds, hydrogen peroxide

## Abstract

Some aquaporins (AQPs) have been recently demonstrated to facilitate the diffusion of hydrogen peroxide (H_2_O_2_) from the producing cells to the extracellular fluid, and their reactive oxygen species scavenging properties have been defined. Nevertheless, the identification of different AQPs acting as peroxiporins, their functional role in eustress and distress, and the identification of antioxidant compounds able to regulate AQP gating, remain unsolved. This study aims to investigate, in HeLa cells: (1) the expression of different AQPs; (2) the evaluation of naringenin, quercetin, (*R*)-aloesaponol III 8-methyl ether, marrubiin, and curcumin antioxidant profiles, via α,α-diphenyl-β-picrylhydrazyl assay; (3) the effect of the compounds on the water permeability in the presence and in the absence of oxidative stress; and (4) the effect of pre- and post-treatment with the compounds on the H_2_O_2_ content in heat-stressed cells. Results showed that HeLa cells expressed AQP1, 3, 8, and 11 proteins. The oxidative stress reduced the water transport, and both pre- and post-treatment with the natural compounds recovering the water permeability, with the exception of curcumin. Moreover, the pre- and post-treatment with all the compounds reduced the H_2_O_2_ content of heat-stressed cells. This study confirms that oxidative stress reduced water AQP-mediated permeability, reversed by some chemical antioxidant compounds. Moreover, curcumin was shown to regulate AQP gating. This suggests a novel mechanism to regulate cell signaling and survival during stress, and to manipulate key signaling pathways in cancer and degenerative diseases.

## 1. Introduction

Hydrogen peroxide (H_2_O_2_) is one of the most abundant and stable reactive oxygen species (ROS) in organisms, produced by superoxide dismutase (SOD) from the superoxide anion, ^•^O_2_^−^ [1,2,3]. Cells can generate H_2_O_2_ by NADPH oxidases (NOX) in the plasma membrane, by oxidative phosphorylation in mitochondria, and by oxidative protein folding in the endoplasmic reticulum [4]. ROS like H_2_O_2_ and 4-hydroxynonenal can exert a physiological effect at low concentrations, acting as intracellular second messengers (signaling molecules), or a cytotoxic effect at high concentrations that may trigger programmed cell death (apoptosis). Thus, the oxidative stress results from the imbalance occurring in the cells between ROS production and scavenging systems or detoxification. Great interest was aroused by the discovery that some aquaporins (AQPs) can facilitate the diffusion of H_2_O_2_ from the producing cells across the plasma membranes to the extracellular fluid [5,6]. The transport of H_2_O_2_ across membranes by specific AQPs has been considered the last milestone in the timeline of hydrogen peroxide discoveries in chemistry and biology [7,8].

AQPs are a family of water channel proteins present in mammals in thirteen isoforms (named from AQP0 to AQP12) with different permeability features and different localizations at cellular and subcellular levels [9,10,11]. To date, AQP3, AQP5, AQP8, AQP9 were found to facilitate H_2_O_2_ diffusion [6,12,13,14,15,16,17,18,19], even though experimental evidence suggests that all water-permeable AQPs can act as peroxiporins, but with different H_2_O_2_ permeabilities: aquaammoniaporins > orthodox aquaporins > aquaglyceroporins [20]. Furthermore, AQP11, an AQP localized in the endoplasmic reticulum, shows a major role in preventing glucose-induced oxidative stress in kidney proximal tubules, but so far, it has not been directly demonstrated to play a role in H_2_O_2_ diffusion [21].

Physiological role for AQP-mediated transmembrane diffusion of hydrogen peroxide in mammalian cells was demonstrated for some AQPs. The direct experimental evidence for AQP-mediated facilitated transmembrane diffusion of H_2_O_2_ across the yeast plasma membrane was obtained by using ROS-sensitive fluorescent dye [12]. The results obtained show that addition of H_2_O_2_ to yeast cells expressing human AQP8 and plant AtTIP1;1, AtTIP1;2, but not hAQP1, significantly increased the intracellular fluorescence. Successively, AQP3 was also demonstrated to mediate hydrogen peroxide uptake and to regulate downstream intracellular signaling [15]. The role of AQP3 in the transmembrane transmission of H_2_O_2_ signals was confirmed by Hara-Chikuma et al. [16]. In detail, the authors demonstrated that AQP3-mediated H_2_O_2_ transport is required for chemokine-dependent T cell migration during the immune response in mice. The involvement of AQP3 in H_2_O_2_ entry and the related cell signaling in cancer cells suggest that AQP3 may represent a new potential therapeutic target for cancer treatment [22,23]. More recently, H_2_O_2_ entry through AQP3 was found to be crucial for wound healing and innate immune function [18].

The expression of rat AQP5 in the *Saccharomyces cerevisiae* model has shown that it also has the property to transport H_2_O_2_, thus modulating the cell response to oxidative stress status [19].

After Miller’s study, AQP8 involvement in H_2_O_2_ transport was also confirmed by Sitia’s group using HeLa cells expressing the fluorescent H_2_O_2_ sensor HyPer [24]. Moreover, they found that AQP8 silencing inhibited not only the H_2_O_2_ entry, but also the phosphorylation of downstream proteins induced by EGF. Further studies showed that different cellular stress conditions reversibly inhibit the permeability of AQP8 to both H_2_O_2_ and water. Neither H_2_O_2_ nor water uptake is impaired in stressed cells expressing a mutant C53S AQP8. Cells expressing this mutant do not accumulate intracellular ROS, and are more resistant to stress-induced growth arrest and death [14].

The involvement of AQP9 in H_2_O_2_ transport was also clearly demonstrated by using Chinese hamster ovary (CHO)-K1 cells with an enforced expression of human AQP9, human AQP9 knockdown HepG2 cells and cells from AQP9 null mice [17]. Recently, we demonstrated the aquaporin-mediated water and H_2_O_2_ transport involvement in normal human spermatozoa functioning [25]. Sperm cells show water and H_2_O_2_ permeability, which was reversibly inhibited by heat stress and the AQP inhibitor HgCl_2_. Reduced functionality is observed in patients with compromised basal semen parameters, suggesting that AQPs are involved in both volume regulation and ROS elimination. 

To sum up, it is clear that AQPs may have a beneficial effect in oxidative stress through a ROS scavenging mechanism, even if some AQP aspects remain unsolved, such as the identification and localization of different AQPs acting as peroxiporins, and their functional role in eustress and distress. In this scenario, the identification of antioxidant compounds able to regulate AQP gating could have a relevant role in understanding the AQP function mechanism. The last issue is the most intriguing, considering that so far, few potential AQP modulators have been identified to date, and their activity sometimes questioned. However, experimental evidence supports AQPs as possible “druggable” proteins [26,27].

Therefore, the study herein presented is aimed (1) to select some natural structurally unrelated compounds, endowed with free radical scavenging (FRS) activities (α,α-diphenyl-β-picrylhydrazyl, DPPH, assay), useful for studying AQPs; (2) to evaluate the expression of mRNA and proteins of different AQPs in HeLa cells by RT-PCR and immunoblotting; (3) to evaluate the water permeability using stopped-flow light scattering method, and the gating of AQPs in presence and in the absence of oxidative stress; and (4) to evaluate the H_2_O_2_ levels in heat-stressed HeLa cells in the presence of the selected antioxidant compounds, measured by a fluorescence method.

## 2. Results

### 2.1. Compound Selection and Free Radical Scavenging (FRS) Activity

**Figure 1:**
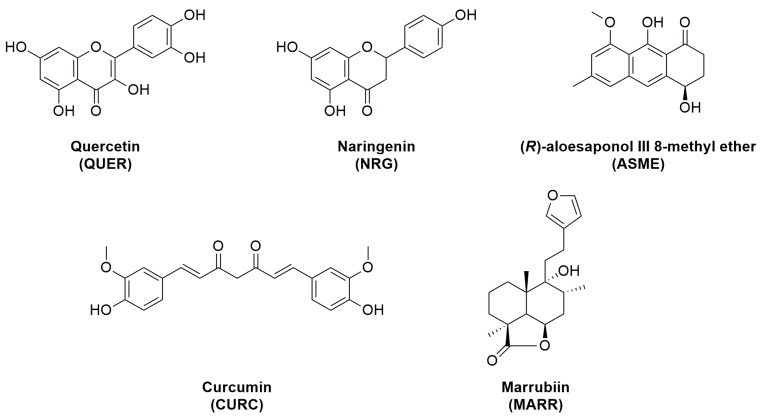
Chemical structures of quercetin (QUER), naringenin (NRG), (*R*)-aloesaponol III 8-methyl ether (ASME), curcumin (CURC), marrubiin (MARR).

The final aim of this work is to identify compounds able to interact with AQP, and to understand, if possible, their action mechanisms. On the basis of our previous experience, we consider five not-structurally related [28,29] natural compounds, belonging to different chemical classes, i.e., quercetin (QUER), naringenin (NRG), (*R*)-aloesaponol III 8-methyl ether (ASME), curcumin (CURC), marrubiin (MARR), (Table 1, Figure 1). 

As a first screening, we performed the DPPH assay, commonly used to determine FRS activity of natural compounds [45]. Moreover, this assay can be helpful in lead-finding of novel antioxidants. DPPH gives rise to a stable radical able to remove a hydrogen atom from the antioxidant (ArOH) that itself becomes a radical, leading to a decrease in the absorbance value (λ = 517 nm):

R**^.^**+ ArOH → RH + ArO (1)

**Figure 2:**
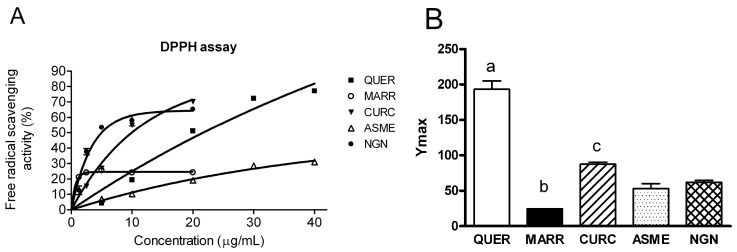
Antioxidant activity determination of quercetin (QUER), marrubiin (MARR), curcumin (CURC), ASME, and naringenin (NRG) by a α,α-diphenyl-β-picrylhydrazyl (DPPH). DPPH assay was performed as described in Materials and Methods. (**A**) Free radical scavenging activity of the compounds was determined as a function of increasing compound concentration. The curves were obtained by fitting the experimental points with computerized least-square regression (see Materials and Methods). The symbols represent means ± SEM of three different experiments. When not shown, SEM were within the symbol area; (**B**) Bars represent the Ymax constant values, obtained as described above. Values are means ± SEM of 3–6 different experiments. (**a**) *p* < 0.05 vs. MARR, CURC, ASME, NRG; (**b**) *p* < 0.05 vs. CURC, ASME, NRG; (**c**) *p* < 0.05 vs. ASME, NRG (ANOVA followed by Newman–Keuls’ *Q* test).

In this work, we evaluate the FRS properties of compounds reported in Figure 1, using QUER as positive control (IC_50_ = 2.1 ± 0.1) [46]. In details, stock solutions of NRG, QUER, ASME, CURC and MARR in methanol were prepared into a range of 1.25–20 µg/mL or 1.25–40 µg/mL, depending on compound solubility, added to a methanolic solution of DPPH, and the absorbance of the resulting solutions evaluated. Results showed a non-linear regression feature of the experimental points (Figure 2A). The maximal FRS activity, Ymax, was obtained by fitting the data with the one phase exponential association (see Materials and Methods) and showed in Figure 2B. Ymax was significantly higher for QUER compared to other compounds tested. The FRS activity order was the following: QUER >> CURC >NRG ≅ ASME > MARR (Figure 2B).

### 2.2. AQP1, 3, 8, 9, and 11 mRNA Are Expressed in HeLa Cells

**Figure 3:**
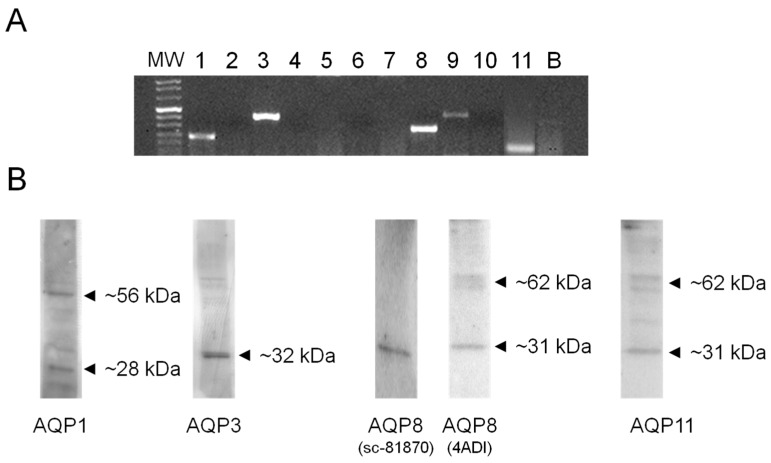
Aquaporin (AQP) mRNA (**A**) and protein (**B**) expression in HeLa cells. (**A**) RT-PCR of total RNA was performed by using specific primers listed in Table 1. Gel electrophoresis showed specific PCR products for AQP1 (229 bp band), AQP3 (414 bp band), AQP8 (282 bp band), AQP9 (432 bp band), and AQP11 (141 bp). Similar results were obtained from at least three different RNA extracts. MW, molecular weight marker. B, negative control; (**B**) Blots representative of three were shown. Lanes were loaded with 30 µg of proteins, probed with affinity purified antibodies, and processed as described in Materials and Methods. Major bands of about 28 kDa (monomer) and 56 kDa (dimer) were observed for AQP1. A single band of about 32 kDa was observed for AQP3 and 8 (using sc-81870 antibody). Major bands of about 31 kDa (monomer) and 62 kDa (dimer) were observed for AQP8 (using 4ADI antibody) and AQP11.

First, we explored by RT-PCR the expression of AQP1-11 mRNA in HeLa cells. AQP1, 3, 8, 9, and 11 transcripts were expressed (Figure 3A). Gel electrophoresis showed single bands of the expected size of the amplified cDNA fragments: 229 bp for AQP1, 414 bp for AQP3, 282 bp for AQP8, 432 bp for AQP9, and 141 bp for AQP11. Similar results were obtained from at least three different RNA extracts. 

### 2.3. AQP1, 3, 8, and 11, but Not AQP9, Protein Are Expressed in HeLa Cells

The expression of AQP proteins in HeLa cells was studied by immunoblotting using affinity pure antibodies. The results indicated that AQP1, 3, 8, and 11 were expressed, while AQP9 protein was not found (Figure 3B). Immunoblots showed major bands of about 28 kDa (monomer) and 56 kDa (dimer) for AQP1. A single band of about 32 kDa was observed for AQP3 and 8 (using Santa Cruz sc-81870 antibody). Major bands of about 31 kDa (monomer) and 62 kDa (dimer) were observed for AQP8 (using Alpha Diagnostic antibody) and AQP11. Similar results were obtained from at least three different experiments. No specific bands were observed when the blots were probed with anti-AQP9. The specificity of the reactions was previously characterized, and checked in experiments performed by incubating the blots with pre-immune rabbit serum .as stated in Materials and Methods.

### 2.4. Effect of Antioxidant Compounds on the Water Permeability in the Presence and in the Absence of Oxidative Stress

**Figure 4:**
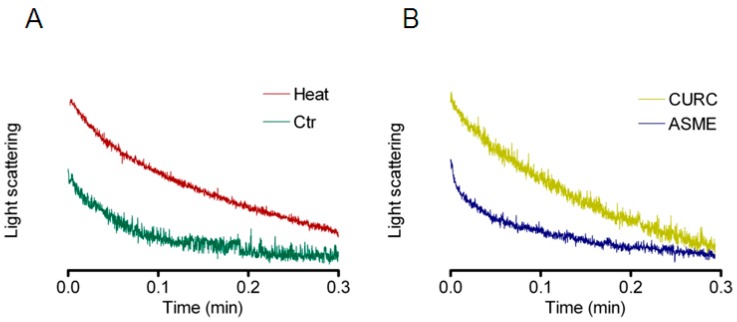
Representative traces of stopped-flow osmotic water permeability measurements obtained from HeLa cells. (**A**) Normal (Ctr) and heat-stress (Heat) conditions; (**B**) effect of pre-treatment with curcumin (CURC) and ASME in heat-stress conditions. HeLa cells were treated as reported in Materials and Methods, and then exposed to a 150 mOsm osmotic gradient.

**Figure 5:**
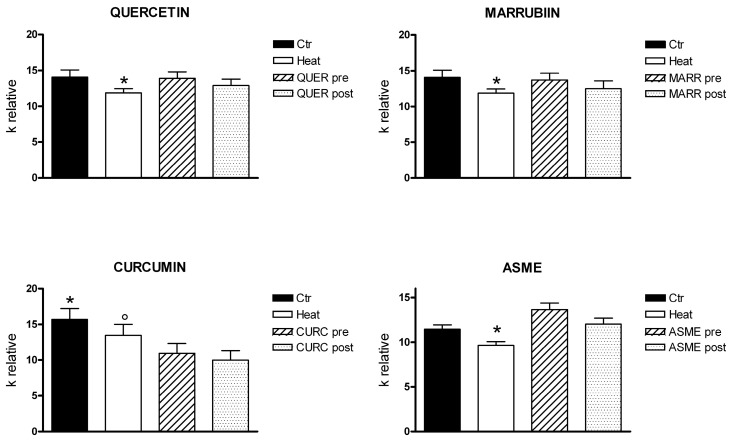

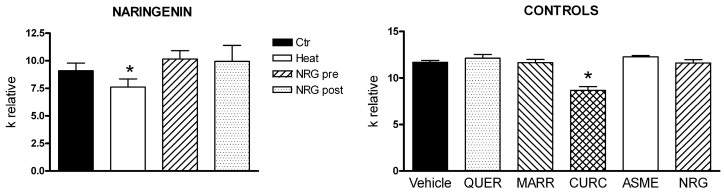
Effect of naringenin (NRG), ASME, quercetin (QUER), marrubiin (MARR), and curcumin (CURC) on the water permeability of HeLa cells in normal and heat-stress conditions. HeLa cells were exposed to a 150 mOsm osmotic gradient in four different conditions: (1) untreated cells (Controls, Ctr); (2) cells treated at 42 °C for 3 h (heat-stressed, Heat); (3) heat-stressed cells pre-treated with the antioxidants compounds (NRG, ASME, QUER, MARR, CURC pre); (4) heat-stressed cells post-treated with the antioxidants compounds (NRG, ASME, QUER, MARR, CURC post). The effect of NRG, ASME, QUER, MARR, CURC on water permeability independently on their antioxidant properties was measured in HeLa cells treated at 21 °C for 3 h with single compounds at 20 µM final concentration. Bars represent the osmotic water permeability of HeLa cells expressed as *k* relative. Values are means ± SEM of 4–15 single shots for each of 4–6 different experiments. NRG, ASME, QUER and MARR: * *p* < 0.05 vs. Ctr, pre and post (ANOVA for repeated measures, followed by Newman–Keuls’ *Q* test). CURC: * *p* < 0.05 vs. Heat, pre and post; ° *p* < 0.05 vs. pre and post (ANOVA for repeated measures, followed by Newman–Keuls’ *Q* test). CONTROLS: * *p* < 0.05 vs. vehicle (ANOVA followed by Dunnett *t* test).

HeLa cells’ exposure to a hypotonic environment caused rapid swelling. The resulting decrease in scattered light intensity was analyzed by a one phase exponential decay equation, and the initial rate constants *k* obtained (Figure 4). Then, to study the effect of oxidative stress on osmotic water permeability, we decided to use heat shock as well-known cell stressor [47]. Heat-treated cells exhibited a significant decrease in water transport when exposed to a hypotonic gradient (Figure 4 and Figure 5). The presence of QUER, MARR, ASME, or NRG during heat treatment prevented the water permeability decrease (Figure 4B and Figure 5). After heat treatment, the incubation of the cells for 30 min with the same compounds restored the water permeability (Figure 5). On the contrary, CURC did not protect nor restore the water permeability of heat-treated cells. 

Water permeability experiments were also performed in HeLa cells incubated at room temperature (in the absence of oxidative stress) in the presence of QUER, MARR, CURC, ASME, and NRG. Unexpectedly, CURC significantly inhibited the water permeability, while other compounds were ineffective (Figure 5, CONTROLS).

### 2.5. Effect of Antioxidant Compounds on the H_2_O_2_ Content in Heat-Stressed HeLa Cells

**Figure 6:**
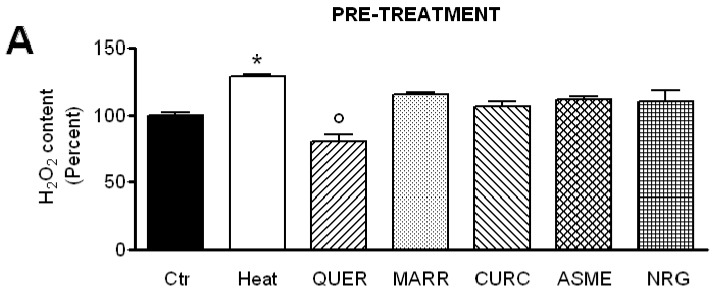

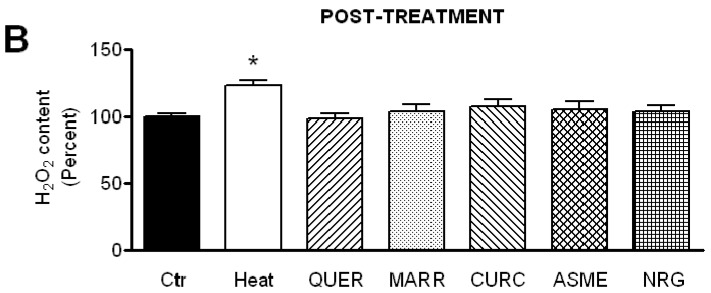
Effect of pre- and post-treatment with quercetin (QUER), marrubiin (MARR), curcumin (CURC), ASME and naringenin (NRG) on the H_2_O_2_ content in heat-stressed HeLa cells. (**A**) Pre-treatment: hydrogen peroxide (H_2_O_2_) was measured using the CM-H2DCFDA reagent (see Materials and Methods) in normal (Ctr) and heat-stressed condition in the presence or in the absence (Heat) of different compounds (20 µM final concentration); (**B**) Post-treatment: H_2_O_2_ was measured in HeLa cells in the following conditions: normal (Ctr), heat-stressed (Heat) and heat-stressed cells post-treated with the antioxidants compounds (20 µM final concentration). Bars represent the H_2_O_2_ content in HeLa cells expressed as percent. Values are means ± SEM of 4 different experiments. * *p* < 0.05 vs. Ctr, QUER, MARR, CURC, ASME, NRG; ° *p* < 0.05 vs. Ctr, Heat, MARR, CURC, ASME, NRG (ANOVA followed by Newman–Keuls’ *Q* test).

The effect of pre- and post-treatment with the antioxidant compounds on the H_2_O_2_ content in heat-stressed HeLa cells was tested by using the CM-H2DCFDA fluorescent probe. Results in Figure 6 show that both pre-treatment and post-treatment with the different compounds reduced the H_2_O_2_ content in heat-stress condition in a statistically significant manner. Moreover, in the pre-treatment condition, QUER was demonstrated the most effective in reducing the H_2_O_2_ intracellular levels even lower than those of control cells.

## 3. Discussion

As a part of our on-going research, we selected compounds of Figure 1 in order to understand the structural features responsible for the interaction with AQPs. We selected five antioxidant compounds which are characterized by different scaffolds, and potentially, different aromatic substitutions. As a first step of our work, we evaluate their FRS properties by a chemical assay (DPPH) widely used as primary screening of antioxidant activity of natural compounds. All compounds showed FRS activity, even with different profiles, with the only exception of MARR endowed with poor FRS properties. We decided to further investigate all compounds to understand their beneficial role in counteracting the oxidative stress. 

Firstly, we demonstrated in HeLa cells the expression of multiple AQPs at both mRNA and protein level: AQP1, 3, 8, and 11 (Figure 3). Among these, AQP3 and AQP8 were found to facilitate the H_2_O_2_ transmembrane transport in different experimental conditions, whereas AQP11 was suggested to have a peroxiporin function, though not directly demonstrated [12,14,15,16,18,21,22,23,24]. Then, we have studied the effect of some antioxidant compounds on the osmotic water permeability of HeLa cells in the presence of oxidative stress condition. Cellular oxidative stress was found to inhibit the permeability to water, as previously observed for AQP8 [14,24,25]. The presence of QUER, NRG, ASME, and MARR, during or after heat-treatment, was able to prevent or restore the water permeability decrease (Figure 4 and Figure 5). Actually, water movement changes could be considered representative of an H_2_O_2_ flux variation [14,25]. A different behavior was evidenced for CURC (Figure 4B and Figure 5). The effect of the compounds was also tested in the absence of oxidative stress, in the hypothesis of their possible direct channel modulation. Surprisingly, CURC significantly inhibited the water permeability, while other compounds were ineffective (Figure 5, CONTROLS).

The antioxidant efficacy of all compounds was then evaluated by measuring the content of H_2_O_2_ in heat-stressed cells. Pre-treatment and post-treatment with all compounds significantly reduced the H_2_O_2_ content in heat-stressed cells. Moreover, in the pre-treatment condition, QUER resulted in the most effective in reducing the H_2_O_2_ intracellular levels, even lower than those of control cells (Figure 6). It is worth noting that also in the DPPH assay, QUER showed relative high potency in comparison to other compounds (Figure 2).

The results are not surprising, in light of recent findings on the beneficial effect of polyphenols, and phytochemical compounds in general, in modulating AQPs gene expression (for a complete review see [48]). Particularly, Kumar et al. found that QUER treatment of streptozotocin-induced diabetic rats prevents the retina neurodegeneration, at least in part by normalizing the expression of AQP4 (inhibition) [49], even if it is not clear if this effect derives from its antioxidant activity or by preventing retinal edema. Conversely, QUER increased AQP5 expression, and decreased oxidative stress and inflammation induced by radiation exposure, in impaired salivary secretion by exposure to radiation [50]. This result is consistent with the function as peroxiporin of AQP5 recently demonstrated [19].

Polyphenols have been shown to modulate AQPs expression, but so far, there was no data about their effects on the osmotic water permeability, or on the direct interaction with AQP/AQPs [48]. The results herein presented, suggest that not only the polyphenols QUER and NRG, but also ASME and MARR, are able to normalize the water permeability, which in turn determines the removal of H_2_O_2_ and oxidative stress resolution. It has been shown that oxidative stress conditions inhibit AQP8-mediated H_2_O_2_ scavenging, and that, in a vicious circle, contribute to worsening the condition [14]. Neither H_2_O_2_ nor water uptake is impaired in stressed cells expressing a mutant C53S AQP8 that do not accumulate intracellular ROS, and are more resistant to stress-induced growth arrest and death.

As regards CURC, we observed a considerably different behavior compared with other substances tested. Despite that it demonstrated clear antioxidant properties (Figure 2 and Figure 6), CURC did not protect nor restore the AQPs’ permeability properties in heat-treated cells, but showed a direct inhibitory effect on AQP/AQPs (Figure 4B and Figure 5). Few potential AQP inhibitors have been identified to date, and some of them have low specificity and high toxicity (see Hg^2+^ and cysteine-targeting heavy metal-based inhibitors in general) [26]. Accordingly, the identification of a new and non-toxic molecule is of particular interest, since AQPs has been proposed as potential “druggable” targets in several pathophysiological conditions [26]. AQPs’ druggability has been recently demonstrated. Molecular docking computations identified a cavity close complementary of arbidol, berbamine, and tamarixetin (a quercetin metabolite) on the extracellular AQP4 surface [51]. Successively, molecular dynamics and molecular interaction fields have allowed for precise identification of a cavity, close to loop A of the AQP4, used by the above-mentioned compounds as a putative binding site [52]. As a whole, these results strongly suggest the possibility, also, for other AQPs to be bound by drug-like molecules, CURC being a valid candidate. The effect of CURC is seemingly paradoxical, because it has well-documented antioxidant properties, but at the same time, inhibits the functioning of AQPs, that in turn heightens the oxidative stress. It could be speculated that CURC may reduce H_2_O_2_ entry into the cell (AQP-mediated) that is produced by oxidative stress through NOX or dual oxidase extracellular activity, or from circulating ROS. However, the effect of CURC in lowering the H_2_O_2_ intracellular levels in heat-stress conditions (Figure 6) derives to other intracellular antioxidant properties of the compounds: the direct ROS scavenging, the metal chelating, and the modulation of cytoprotective and antioxidant proteins via the expression of the transcription factor nuclear factor-erythroid-2-related factor 2 [53]. The antioxidant, neuro-protective, anti-inflammatory, anti-cancer properties of CURC have been recognized for millennia, and it is commonly used in traditional medicine [54]. It has been demonstrated that there is a downregulation of AQP4 (normalization) after CURC administration in different neurological injury and brain damage models like: traumatic brain injury, hypoxia–ischemic brain damage, hypoxia-hypercapnia-induced brain damage, and spinal cord injury [55,56,57,58]. In mice with intracerebral hemorrhage, CURC dose-dependently decreased AQP4 and AQP9, but not AQP1 expression, through the NF-κB signaling pathway [59]. CURC treatment was also found to downregulate AQP1 in rat choroid plexus cells, useful to reduce cerebrospinal fluid production in some pathophysiological conditions [60], and AQP3 expression in human ovarian cancer cells [61]. However, the biological functions of CURC are far from fully clarified, and sometimes appear conflicting [54]. In conclusion, all the compounds analyzed have confirmed their antioxidant capacity. QUER, NRG, ASME, and MARR are able to protect or restore the AQP-mediated H_2_O_2_ cellular elimination, while CURC seems to prevent H_2_O_2_ cellular entry. The results presented here suggest the possibility to chemically regulate the pore gating of peroxiporins, and provide a new direction to the development of new therapeutic treatments for cancer, degenerative diseases, as well as in ageing.

## 4. Materials and Methods

### 4.1. Cell Culture

HeLa cells were routinely grown in plastic tissue culture flasks using Dulbecco’s modified minimal essential medium high glucose, supplemented with 10% fetal bovine serum, 1% l-glutamine, 1% penicillin and streptomycin, and maintained at 37 °C in a humidified atmosphere of 5% CO_2_, 95% air.

### 4.2. RNA Isolation and RT-PCR

Total RNA was extracted from HeLa cells using QIAzol Lysis Reagent (Qiagen SpA, Milan, Italy), and reverse transcription was performed according to Laforenza et al. [62]. cDNA amplification was performed by GoTaq^®^ Flexi DNA Polymerase (Promega, Milano, Italy), as previously described [62]. The primers used for amplification are listed in the Table 2. The PCR protocol consisted of an initial denaturation of 3 min at 95 °C followed by 35 cycles of denaturation at 96 °C for 30 s, annealing (see TM in Table 1) for 30 s, and extension at 72 °C for 30 s. Reverse transcription was always performed both either in the presence (positive) or in the absence (negative control) of reverse transcriptase enzyme. PCR products were separated on a 3% Nusieve^®^ (2:1) gel agarose, stained with ethidium bromide, and acquired with the Image Master VDS (GE Healthcare, Milano, Italy). The molecular weight of the PCR products was compared with the DNA molecular weight marker VIII (Roche Molecular Biochemicals, Monza, Italy).

### 4.3. Immunoblotting

HeLa cells were treated as previously described [63]. Solubilized proteins (30 µg) were subjected to 12.5% SDS-polyacrylamide gel electrophoresis, and transferred to Hybond-P PVDF membrane (GE Healthcare) by electroelution. The membranes were incubated overnight with anti-AQP1 rabbit polyclonal IgG affinity pure (AQP1-A, 1:800; Alpha Diagnostics Intl. Inc., San Antonio, TX, USA), anti-AQP3 rabbit polyclonal IgG (sc-20811, 1:300; Santa Cruz Biotechnology, Inc., Heidelberg, Germany), anti-AQP8 rabbit polyclonal IgG affinity pure (AQP8-A, 1:500; Alpha Diagnostics Intl. Inc.; sc-81870, 1:500; Santa Cruz Biotechnology, Inc., Heidelberg, Germany, anti-AQP9 mouse polyclonal IgG (sc-74409, 1:300; Santa Cruz Biotechnology, Inc.), and affinity purified rabbit anti-human AQP11 polyclonal antibody (AP5805b, 1:500; Abgent Inc., San Diego, CA, USA), previously characterized [25,64,65,66]. The membranes were washed and incubated for 1 h with goat anti-rabbit IgG antibody, peroxidase conjugated (AP132P; Millipore part of Merck S.p.a., Vimodrone, Italy) or with peroxidase-conjugated mouse IgG from Dakocytomation (P0260), diluted 1:100,000 in blocking solution. The bands were detected with ECL™ Select Western blotting detection system (GE Healthcare). Prestained molecular weight markers (Precision Plus Protein™ Dual Color Standards, #1610374, Bio-Rad, Segrate, Italy) were used to estimate the molecular weight of the bands.

### 4.4. DPPH Assay

The FRS of the extracts was determined by using a DPPH assay [39]. QUER was used as a standard. Briefly, all compounds were dissolved in MeOH, and then serially diluted in the same solvent until the target concentrations were reached. Each compound was tested at different concentrations, in the range allowed by their solubility. FRS was expressed as a percent compared with the reference compound, consisting of 3.9 mL of DPPH solution and 100 µL of methanol. The percent inhibition of the DPPH radical by the test solution was calculated using the following formula:

FRS% = [(Abs control − Abs sample)/Abs control] × 100(2)

The analyses were carried out in triplicate, and results are expressed as mean ± SEM.

### 4.5. Water Permeability Measurements

Osmotic water permeability of the HeLa cells was measured by stopped-flow light scattering method [64,66]. Briefly, the experiments were carried out at room temperature on a stopped flow apparatus (RX2000, Applied Photophysics, Leatherhead, UK) with a pneumatic drive accessory (DA.1, Applied Photophysics) coupled with a Varian Cary 50 spectrometer (Varian Australia Pty Ltd., Mulgrave, Australia). Scattered light intensity with a dead time of 6 ms was recorded at a wavelength of 450 nm. The time course of cell swelling caused by exposure to the hypotonic gradient (150 mOsm/L) was measured for 60 s at the acquisition rate of one reading/0.0125 s. Cells behaved as perfect osmometers, the gradient determined an osmotic water entry, cell swelling, and decreased light scattering. The initial rate constant of cells volume changes (*k*) was obtained by fitting the points of the time course of light scattering with a one phase exponential decay equation, calculated by computerized least squares regression (GraphPad Prism 4.00, La Jolla, CA, USA, 2003). To study the effect of oxidative stress on water permeability and the antioxidant effect of different natural compounds, HeLa cells were divided into four groups: (1) controls, cells left at room temperature (21 °C); (2) heat-stressed cells, cells subjected to heat-shock treatment by placing them in a water thermostatic and shacking bath at 42 °C for 3 h; (3) heat-stressed cells pre-treated, cells incubated in a water thermostatic and shacking bath at 42 °C for 3 h together with the antioxidants compounds NRG, ASME, QUER, MARR, and CURC at 20 µM final concentration (dissolved in methanol); (4) heat-stressed cells post-treated, cells incubated in a water thermostatic and shacking bath at 42 °C for 3 h, and then further incubated at 21 °C for 30 min with the antioxidant compounds. To study the effectiveness of the analyzed compounds to affect the AQP gating independently of their antioxidant properties, HeLa cells were treated in the presence and in the absence of the compounds by incubating at 21 °C for 3 h.

### 4.6. Hydrogen Peroxide Measurements

Hydrogen peroxide levels in HeLa cells were measured by a fluorescence method using the 5-(and-6)-chloromethyl-2′,7′-dichlorodihydro-fluorescein diacetate, acetyl ester reagent (CM-H2DCFDA) (Invitrogen). Briefly, cells were centrifuged at 200 rcf for 5 min. The cell pellet was resuspended in PBS and CM-H2DCFDA reagent was added at 5 mM final concentration and incubated for 1 h at room temperature. Thereafter, cells were centrifuged again, and the pellet resuspended in PBS. Hydrogen peroxide levels were measured in the experimental conditions described above by using a CLARIOstar^®^ microplate reader (BMG LABTECH, Ortenberg, Germany).

### 4.7. Protein Content

The protein content was determined with the Bradford method [67], using bovine serum albumin as standard.

### 4.8. Statistics

All data were expressed as means ± SEM (Standard Error Mean). The significance of the differences of the means were evaluated by using one-way ANOVA, followed by Newman–Keuls’ *Q* test, or Student’s *t* test. All statistical tests were carried out with GraphPad Prism 4.00, 2003.

## Figures and Tables

**Table 1:** Biological activity, natural sources, and chemical class of the compounds tested.

Compound	Chemical Class	Natural Sources	Biological Activity	References
QUER	flavonoid	caper, black chokeberry, onion, tomato and lettuce	antioxidant, anti-inflammatory, anti-obesity, antiviral, antibacterial, anti-cancer and others	[30,31,32,33]
NRG	flavanone	*Citrus* fruits, tomato, pears, *Amygdalus lycioides*	Antoxidant, anti-inflammatory, antimicrobical, anti-cancer and immunomodulating activities	[34,35,36,37,38]
ASME	tetrahydroanthracene	*Eremurus persicus*; *Aloe saponaria*; *Kniphonia foliosa*; *Eremurus chinensis*	Antileishmanial activity	[39]
CURC	phenol	*Curcuma longa*, *Curcuma* species	Antioxidant, anti-inflammatory, antinociceptive, anticancer, immunomodulating properties and others	[33,40,41,42]
MARR	terpenoid	*Marrubium vulgare*, *M.* spicies, *Phlomis bracteosa*, *Leonotis nepetifolia*	antinociceptive, antioxidant, cardioprotective, vasorelaxant, gastroprotective, antispasmodic, immunomodulating, antioedematogenic, analgesic, antidiabetic properties	[43,44]

Abbreviations: QUER, quercetin; NRG, naringenin; ASME, (*R*)-aloesaponol III 8-methyl ether; CURC, curcumin; MARR, marrubiin.

**Table 2:** Primer sequences used for real time reverse transcription/polymerase chain reaction.

Gene	Primer Sequences		Size (bp)	TM (°C)	Accession Number
*AQP1*	Forward	5′-TTAACCCTGCTCGGTCCTTT-3′	229	60	NM_198098
	Reverse	5′-TTCATCTCCACCCTGGAGTT-3′			
*AQP2*	Forward	5′-CCACACTCCTCTTCGTCTTCTT-3′	552	60	NM_000486
	Reverse	5′-CCCAGTGGTCATCAAATTTGCC-3′			
*AQP3*	Forward	5′-TTTGCTACCTACCCCTCTGG-3′	414	60	NM_004925
	Reverse	5′-GGCCAGCTTCACATTCTCTT-3′			
*AQP4*	Forward	5′-GGTCTCCTGGTTGAGTTGAT-3′	346	60	NM_004028
	Reverse	5′-TTGTTTGCTGGGCAGCTTTG-3′			
*AQP5*	Forward	5′-GAGCTGATTCTGACCTTCCA-3′	333	58	NM_001651
	Reverse	5′-AGGCTCATACGTGCCTTTGA-3′			
*AQP6*	Forward	5′-GGGCTGTATGTGTTCTTTGG-3′	326	60	NM_001652
	Reverse	5′-TGGCCAGTTGAGACACTGTT-3′			
*AQP7*	Hs_AQP7_1_SG QuantiTect Primer Assay QT00067592 *		139	60	NM_001170
*AQP8*	Forward	5′-AGGAGAGGTTCTGGAATGCA-3′	282	60	NM_001169
	Reverse	5′-AGTGGAAGTTCCAGTGGTTG-3′			
*AQP9*	Forward	5′-AAGCTTCAAGCAGAGACTGG-3′	432	60	NM_020980
	Reverse	5′-GGAGCTGGGTATGTTGCAAA-3′			
*AQP10*	Forward	5′-CCCTATCTGTCCCTGAACAA-3′	554	60	NM_080429
	Reverse	5′-CCAGGTTCTGGCACATTAAC-3′			
*AQP11*	Forward	5′-GGCTGCACTCATCACCTTTT-3′	141	58	NM_173039
	Reverse	5′-GGAGCCAGCCAGTATACTAT-3′			

TM, Melting Temperature; *, from Qiagen.

## Abbreviations

H_2_O_2_	Hydrogen peroxide
ROS	Reactive oxygen species
SOD	Superoxide dismutase
NOX	NADPH oxidases
AQP	Aquaporin
CM-H2DCFDA	5-(and-6)-chloromethyl-2′,7′-dichlorodihydrofluorescein diacetate, acetyl ester
ASME	(*R*)-aloesaponol III-8 methyl ether
NRG	Naringenin
QUER	Quercetin
MARR	Marrubiin
CURC	Curcumin
FRS	Free radical scavenging
DPPH	α,α-diphenyl-β-picrylhydrazyl
SEM	Standard error mean
